# The Evolution of FAP-Targeted CAR T-Cell Therapy in Solid Tumors: From Immunotherapy to Immunotheranostic Applications

**DOI:** 10.3390/ijms27167425

**Published:** 2026-08-19

**Authors:** Hugo Boutier, Anja Feldmann, Michael Bachmann

**Affiliations:** 1 Department of Radioimmunology, Institute of Radiopharmaceutical Cancer Research, Helmholtz-Zentrum Dresden-Rossendorf (HZDR), 01328 Dresden, Germany; 2 National Center for Tumor Diseases (NCT), NCT/UCC Dresden, 01307 Dresden, Germany; 3 German Cancer Consortium (DKTK), Partner Site Dresden, and German Cancer Research Center (DKFZ), 69120 Heidelberg, Germany; 4 Faculty of Medicine and University Hospital Carl Gustav Carus, TU Dresden, 01307 Dresden, Germany

**Keywords:** fibroblast activation protein (FAP), tumor microenvironment, CAR T-cell, adapter CAR platform, FAPI, targeted radioligand delivery, theranostics, immunotheranostics

## Abstract

Chimeric antigen receptor (CAR) T-cell therapy has revolutionized the treatment landscape of hematologic malignancies but has demonstrated limited efficacy in solid tumors, mainly due to the complex and immunosuppressive tumor microenvironment (TME). Among the strategies to overcome this challenge, targeting the tumor stroma rather than the tumor cells themselves has gained increasing interest. In this context, fibroblast activation protein alpha (FAP), a cell surface protease overexpressed by cancer-associated fibroblasts, represents a promising target. With the aim of remodeling the TME, enhancing immune infiltration, and suppressing tumor growth, numerous FAP-directed CAR T-cell therapies have been developed in the last decade, leading to the clinical translation of two candidates. To improve the flexibility and safety profile of CAR T-cell therapies, several groups have designed more controllable and modular approaches, including adapter CAR T-cell systems, which enable on-demand activation of effector cells through the administration of an adapter molecule. In parallel, the development of FAP-targeted radiotracers, particularly FAP inhibitors (FAPIs), has enabled high-contrast imaging of solid tumors and introduced attractive opportunities for radioligand therapy. The convergence of these advances has given rise to immunotheranostic strategies that integrate CAR T-cell immunotherapy and radioligand delivery within a unified framework. This review traces the evolution of FAP-directed CAR T-cell strategies, from conventional designs to adapter-based and theranostic platforms, and examines how modular adapters bring immunotherapy and radioligand delivery together within a single immunotheranostic framework, across preclinical and clinical settings.

## 1. Introduction

Despite major advances in both cancer diagnosis and treatment, cancer remains a major global health challenge and the number of cancer cases is expected to grow significantly by 2050 [1]. This emphasizes the critical need for the design of innovative tools for the early and effective detection of tumors, as well as the development of novel therapeutic strategies. In recent decades, various immunotherapeutic strategies have emerged as promising approaches for improving cancer treatment [2]. Among these, adoptive cell therapies (ACTs) aim to enhance the body’s natural anticancer responses by reprogramming patient-derived (autologous approach) or healthy donor-derived (allogeneic approach) immune cells ex vivo. This concept gave rise to T-cell-based therapies, including chimeric antigen receptor (CAR) T-cell therapy [3,4].

In 1989, Zelig Eshhar and his team described the idea of T-bodies, which are genetically modified T-cells expressing artificial, antibody-derived receptors for specific targeting of tumor cells [4]. Following this work, Eshhar et al. engineered the first CAR in 1993 (nowadays referred to as first-generation CAR) by combining an extracellular scFv targeting a specific antigen with the intracellular signaling subunit CD3ζ of the T-cell receptor [3]. In contrast to unmodified T-cells, CAR T-cells specifically recognize antigens on the surface of tumor cells without the need for antigenic peptide presentation via major histocompatibility complex (MHC) molecules, leading to T-cell activation and antitumor response. Since their introduction, CAR T-cell therapies have demonstrated remarkable efficacy in hematologic malignancies, leading to the approval of seven CAR T-cell products for these indications since 2017 [5,6,7].

Despite outstanding efficacy in hematologic malignancies, CAR T-cell therapies have faced several challenges. Due to the limited controllability of conventional CAR designs, CAR T-cells may remain permanently active following administration in patients, potentially leading to substantial adverse events. Furthermore, their therapeutic efficacy in solid tumors has remained modest, largely due to antigen heterogeneity, which promotes antigen escape, as well as the immunosuppressive tumor microenvironment (TME), which actively supports tumor progression and impairs immune cell effector functions [8,9]. These limitations have driven the design of modular immunotherapeutic approaches, including adapter CAR systems, which improve controllability, specificity, and functional versatility [10,11]. In contrast to conventional CAR designs, adapter CARs indirectly interact with a tumor-associated antigen (TAA) through an adapter molecule, which acts as an ON/OFF switch.

Unlike hematologic malignancies, solid tumors develop within a highly complex, heterogenous, and active microenvironment, which limits the therapeutic efficacy of cancer treatments. Among the components of the TME, cancer-associated fibroblasts (CAFs) account for up to 90% of the cellular mass and play an essential role in tumor development, influencing extracellular matrix remodeling, angiogenesis, tumor infiltration, as well as immune cell recruitment and differentiation [12,13,14]. A key marker of CAFs is the fibroblast activation protein alpha (FAP), a type II transmembrane serine protease actively involved in tumor proliferation, migration, and invasion [15,16,17]. Given its overexpression in approximately 90% of epithelial tumors and minimal expression in healthy adult tissues, FAP has become a promising, broadly applicable target across multiple solid tumor types [18,19]. In this context, FAP has gained increasing attention as a target for both conventional CAR T-cell therapy [16,20] and radioligand-based approaches [21,22,23,24], including diagnostic imaging and targeted radioligand therapy. The convergence of these advances has given rise to immunotheranostic strategies that integrate CAR T-cell immunotherapy and radioligand delivery within a unified platform [25,26,27]. This review provides an overview of the recent advances in FAP-directed CAR T-cell strategies, from conventional to adapter-based and theranostic approaches, with insights from both preclinical and clinical studies. Whereas previous reviews have addressed FAP biology, conventional FAP-directed CAR T-cell therapy, or FAP-targeted radiopharmaceuticals as largely separate fields, the present review focuses specifically on their convergence. We examine how modular adapter CAR platforms allow a single FAP-directed molecule to serve as a diagnostic and/or radiotherapeutic agent as well as a switch for CAR T-cell redirection.

## 2. Advances in CAR T-Cell Therapy: From Conventional to Adapter-Based Approaches

### 2.1. Conventional CAR T-Cell Therapies: Clinical Success and Challenges

#### 2.1.1. CAR Design and Approval of CAR Products for Hematologic Malignancies

Over the past decades, CAR T-cell therapy has shown outstanding efficacy for the treatment of hematologic malignancies including lymphoma, leukemia, and multiple myeloma. This led to the clinical approval of several CAR products from 2017 [5,6]. Kymriah (tisagenlecleucel), which is an autologous CD19-targeted CAR T-cell product, was the first CAR-based therapy authorized by the FDA. This approval followed a multicenter Phase II study assessing its safety and efficacy in pediatric and young adult patients with relapsed or refractory B-cell acute lymphoblastic leukemia [28]. The trial reported an overall remission rate of 81% after a single infusion, associated with long-term CAR T-cell persistence. Although manageable, high-grade toxicities such as cytokine release syndrome (CRS) and neurotoxicity were observed. In 2018 and 2022, Kymriah received additional approval for adults with relapsed/refractory large B-cell lymphoma or follicular lymphoma following further clinical studies, respectively [6,29,30]. Following the initial approval of tisagenlecleucel in 2017, six additional anti-CD19 and anti-B-cell maturation antigen (BCMA) CAR-based products have also received regulatory approval.

Since their introduction in the 1990s, multiple generations of CAR T-cells have been designed to enhance their efficacy, persistence, and safety. Due to the presence of only one intracellular domain (CD3ζ), first-generation CAR T-cells were associated with poor proliferation and persistence as well as limited cytokine production (e.g., IL-2), leading to low antitumor efficacy in vivo [5]. To overcome this issue, second-generation CARs, which include an additional costimulatory signaling domain such as CD28 or 4-1BB, were developed. This design leads to a stronger, longer-lasting activation and persistence of CAR T-cells [5,31]. To date, all of the approved CAR T-cell therapies and the most advanced clinical trials involve second-generation CAR T-cells [5,6]. To further enhance CAR T-cell efficacy, third-generation CARs were designed to incorporate two costimulatory domains (e.g., CD28 and 4-1BB or OX40) into the same construct. Although preclinical studies suggest that third-generation CAR T-cells may display higher efficacy and persistence than second-generation CAR T-cells, they may also be associated with severe side effects and rapid exhaustion [5,31]. Fourth-generation CAR T-cells, also known as T-cells redirected for universal cytokine-mediated killing (TRUCKs) or armored CAR T-cells, contain an additional transgene alongside a second-generation CAR backbone. Upon antigen recognition, TRUCKs secrete transgenic immunostimulatory cytokines, which can therefore enhance expansion, persistence and overall immune responses [5,31]. Finally, fifth-generation CAR constructs are also based on second-generation CARs and additionally contain a cytokine-inducible receptor that stimulates the JAK-STAT pathway. These signals not only sustain CAR T-cell activity but also lead to enhanced potency and reduced exhaustion [5].

#### 2.1.2. Challenges Associated with Conventional CAR T-Cell Therapies

Despite remarkable efficacy in hematologic malignancies, conventional CAR T-cells have also been associated with significant risks and limitations. These therapies may indeed cause severe or life-threatening side effects such as cytokine release syndrome (CRS) and immune effector cell-associated neurotoxicity syndrome (ICANS), which result from sustained antigen recognition, CAR T-cell activation, and the release of substantial amounts of cytokines such as IFNγ, GM-CSF, or IL-6, leading to systemic inflammation [32,33,34]. Furthermore, the expression of tumor-associated antigens (TAAs) on malignant cells and healthy tissues can result in on-target/off-tumor toxicity, potentially causing significant damage to non-malignant cells [35]. In this context, the CAR design, including its generation, antigen-binding affinity, and target selection, is critical for ensuring both safety and therapeutic success.

Beyond safety concerns, CAR T-cell therapies have demonstrated limited efficacy in solid tumors, mainly due to antigen heterogeneity and TME-mediated immunosuppression, which impairs CAR T-cell trafficking and persistence. Since CAR T-cell therapies generally target a single antigen, they are particularly vulnerable to treatment resistance or relapse driven by tumor antigen heterogeneity and antigen loss [33,36,37].

Together, these safety issues and the limited adaptability of conventional CAR designs have driven the development of modular CAR platforms, aimed at improving flexibility, specificity, and functional versatility.

### 2.2. Adapter CAR T-Cell Systems: Expanding Functional Flexibility and Controllability

**Figure 1:**
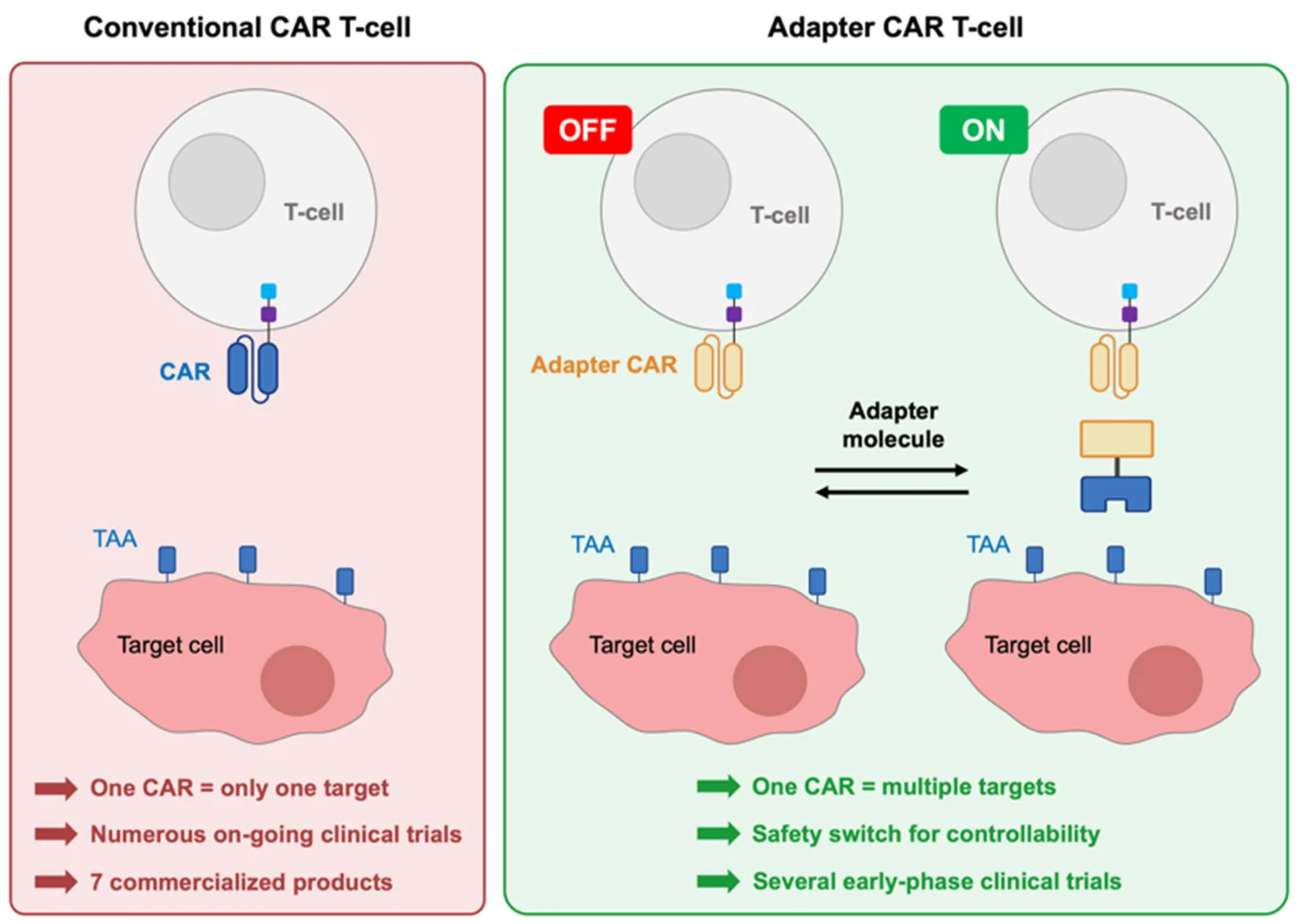
Conventional vs. adapter CAR T-cells. While conventional CAR T-cells express a CAR that directly recognizes a specific target, adapter CAR T-cells express a modular CAR that does not recognize any TAA. Instead, the adapter CAR interacts with an adapter molecule, which binds to both the adapter CAR T-cells and target cells, thereby leading to effector cell activation and selective target cell lysis. With the adapter CAR approach, CAR T-cells do not need to be re-engineered to target other markers and their activity only relies on the presence of adapter molecules.

To address the challenges observed with conventional CAR T-cells, multiple adapter CAR platforms, also known as switchable or modular CAR systems, have been designed and studied in recent years [10,11]. In these systems, T-cells are engineered to express a universal CAR that does not directly interact with a TAA. Instead, adapter CAR T-cells are redirected to TAA-expressing cells through a bridging molecule, also called adapter molecule or target module (TM). Adapter CAR T-cell activation therefore specifically relies on the presence of such an adapter molecule, which on the one hand interacts with the adapter CAR, and, on the other hand binds to the TAA (Figure 1).

Decoupling antigen recognition from CAR-mediated functionality provides several advantages. First, it enables controllability and enhanced safety, because CAR-mediated activity only relies on the presence of an adapter molecule, therefore acting as an ON/OFF switch (Figure 1). This ON/OFF switch relies on several key parameters, including the adapter’s pharmacokinetic properties, as well as its valency and affinity to both the target and modular CAR [10,11]. Adapter CAR systems also have the potential to improve treatment efficacy and target specificity as they enable the use of several adapter molecules with diverse target specificities that can be administered sequentially or in combination without the need to re-engineer the CAR. In this context, they allow for the establishment of logic gating strategies such as OR, NOR, AND and NAND gates (e.g., [38,39,40]). This flexibility is particularly advantageous for addressing tumor heterogeneity and is especially relevant when targeting antigens that are not exclusively expressed on malignant cells. In addition to flexibility and controllability, adapter CAR platforms allow for the local delivery of payloads such as fluorescent dyes or radionuclides via adapter molecules (e.g., [27,41,42]). Notably, the delivery of a suitable radionuclide allows for the implementation of radio-/immunotheranostic approaches, in which radiolabeled adapters can be used for tumor imaging, targeted radionuclide therapy, and treatment monitoring, while also enabling CAR-based immunotherapy. In this context, the fibroblast activation protein has emerged as a promising immunotheranostic target in solid tumors [25,26,27].

To date, several adapter CAR systems including the UniCAR, RevCAR, PNE-CAR, ACTR, and P329G CAR platforms have reached early-phase clinical trials (e.g., NCT04230265, NCT05949125, NCT04450069, NCT03189836, NCT05199519) [43,44,45,46,47]. Adapter CAR therapies have been primarily evaluated in hematologic malignancies, with only limited studies in solid tumors [11]. Overall, administration of adapter CAR T-cells has been associated with manageable toxicities and clinical response [11,43,44,45,46,47]. Despite encouraging initial results, further clinical investigation is required to advance these approaches to later-stage trials and ultimately achieve regulatory approval. Key clinical parameters, such as dosing dependency and scheduling of adapter administration, remain to be fully defined. From a regulatory perspective, the modular nature of these systems introduces additional complexity and raises important questions. To date, it is unclear whether the use of the same CAR T-cell platform with different adapter molecules would require separate approvals for each adapter, or if the approval of the CAR T-cell product could be extended to multiple adapters. As with conventional CAR T-cells, future developments may involve allogeneic, off-the-shelf platforms or in vivo generation of adapter CAR T-cells, both of which offer potential advantages in scalability and accessibility but raise additional considerations regarding safety, control, and regulatory requirements.

## 3. Fibroblast Activation Protein

### 3.1. Biology of Human Fibroblast Activation Protein

FAP is a type II transmembrane serine protease that belongs to the dipeptidyl peptidase (DPP) subfamily and prolyl peptidase family. This family includes related proteases such as DPPIV, DPP7, DPP8, DPP9, and prolyl carboxypeptidase [17,20]. FAP shares approximately 52% amino acid sequence identity with DPPIV, reflecting its close structural homology within the dipeptidyl peptidase family and highlighting the need for careful design of selective FAP-specific agents [20]. Despite this similarity, FAP exhibits distinct substrate specificity and functional roles within the TME. A broad range of substrates has been described, including denatured collagens I and III [48,49], α2-antiplasmin [50], fibroblast growth factor 21 (FGF21) [51,52], fibrillin-2 [53], and extracellular matrix protein 1 (ECM-1) [53].

The human FAP protein has a molecular weight of 97 kDa and comprises 760 amino acids, consisting of a 6 amino-acid N-terminal cytoplasmic domain (aa 1-6), a 20 amino-acid transmembrane region (aa 7-26) and a 734 amino-acid long extracellular domain (aa 27-760) [54]. Structurally, FAP exists as a homodimer at the cell surface, and dimerization is required for full catalytic activity [55]. FAP possesses a dipeptidase activity, enabling the hydrolysis of prolyl bonds located two amino acids from the N-terminus of protein substrates. It also exhibits a post-proline endopeptidase activity, often referred to as gelatinase activity [55,56]. The active site of the FAP dimer consists of a catalytic triad composed of serine-624 (Ser^624^), aspartic acid-702 (Asp^702^), and histidine-734 (His^734^) residues, which together mediate substrate cleavage [54,55].

### 3.2. Expression, Role and Rationale for Targeting FAP in Solid Tumors

Under physiological conditions, FAP expression in adult tissues is generally minimal. Transient upregulation is observed during tissue remodeling, wound healing, inflammation and fibrosis, meaning that fibrotic, inflammatory, or healing lesions may constitute FAP-positive sites in patients [13]. In addition, FAP expression has been detected in bone marrow-derived mesenchymal stromal cells [57] and in the alpha cells of the islets of Langerhans [58]. This context-dependent expression pattern defines the therapeutic window of FAP-directed strategies.

As well as being a transmembrane protein, a catalytically active and soluble form of FAP (sFAP), which lacks the 26 amino acids of the cytoplasmic and transmembrane domains, is also expressed in the blood circulation of healthy individuals [59,60,61]. However, its precise physiological role remains unclear. The presence of sFAP in the plasma raises important questions regarding its potential impact on the pharmacokinetics of FAP-directed diagnostic and therapeutic agents, including radiopharmaceuticals or immunotherapies. Circulating sFAP antigens could theoretically bind FAP-directed molecules before they reach the tumor and therefore affect their biodistribution. Interestingly, Javidroozi and colleagues demonstrated that sFAP levels in patients with gynecologic, hematologic, head and neck, lung, upper gastrointestinal, kidney, bladder, and colorectal cancers were significantly lower than in healthy individuals [60]. Moreover, the high tumor-to-background ratios achieved clinically with radiolabeled FAP inhibitors in solid tumors suggest that sFAP does not substantially limit tracer accumulation at the tumor site [21,62]. However, no study has directly quantified adapter or tracer distribution as a function of sFAP concentration, and the behavior of larger, longer-circulating molecules (e.g., antibodies) for which the interaction with plasma sFAP may be longer than for rapidly cleared small-molecule tracers remains uncharacterized.

In the context of solid tumors, surface FAP is overexpressed in more than 90% of epithelial cancers, making it an attractive target for cancer diagnosis and treatment [18]. It is predominantly detected on the surface of cancer-associated fibroblasts (CAFs) within the tumor microenvironment, and to some extent on tumor cells themselves [13,63,64]. In 2019, Kratochwil and colleagues demonstrated FAP expression across up to 28 different tumor entities, including breast, esophageal, pancreatic, prostate, cervical and ovarian cancers, supporting its characterization as a “pan-tumor” marker [19]. High FAP expression has consistently been associated with poor prognosis in several tumor entities, including breast, lung, bladder, colorectal and ovarian cancer [63,65]. Moreover, elevated stromal FAP levels have also been linked with advanced tumor stage, lymph node or distant metastases, and reduced overall survival [63]. These observations support the idea that FAP is not only a stromal and tumoral marker but also a biologically active protein associated with aggressive disease. In this context, recent studies have demonstrated that FAP plays a key role in tumor progression, contributing to tumor proliferation and invasion through several signaling pathways, including PI3K/AKT [66,67], SHH/GLI [66] and RAS/ERK [67], as well as through extracellular matrix remodeling [17] and promotion of angiogenesis [68,69].

In solid tumors, FAP is predominantly expressed on CAFs, which may exert multiple pro-tumorigenic functions. Because CAFs are considered more genetically stable than malignant cells, FAP-directed strategies may be less susceptible to antigen escape [17]. CAFs are nonetheless a heterogeneous population. Single-cell studies have defined functionally distinct subsets broadly categorized as myofibroblastic, inflammatory, or antigen-presenting CAFs [70,71,72]. These subsets differ in distribution and in their tumor-promoting versus tumor-restraining roles [73], and their spatial organization varies across tumor types and stages [74]. Broad stromal depletion may therefore be not uniformly beneficial: in some models, indiscriminate removal of stromal populations accelerated tumor progression [75,76]. Selective depletion of the FAP^+^ compartment, however, has been associated with reduction in immunosuppression and enhanced antitumor immunity [77,78], suggesting that the therapeutic value of FAP targeting depends on which CAF subsets express FAP in a given tumor and on distinguishing tumor-restraining from tumor-promoting stroma. Targeting FAP therefore represents a promising strategy to disrupt the tumor-supportive stroma rather than to achieve broad, indiscriminate stromal depletion.

## 4. FAP-Directed Conventional CAR Therapy: Efficacy and Challenges

### 4.1. Preclinical Evidence and Translational Challenges

#### 4.1.1. Preclinical Evidence

Given its selective expression in the TME of most carcinomas, FAP has become an attractive target for CAR-based immunotherapy [16,18,20]. FAP-targeted CAR therapies aim to indirectly disrupt tumor growth by remodeling the TME, altering stromal support and promoting immune cell penetration. Over the past 15 years, numerous preclinical studies have investigated the potency of FAP-targeted CAR-based therapies using diverse in vitro and in vivo models for a broad range of tumor types, including lung, melanoma, colon, breast, kidney, pancreatic, brain, and liver cancers [79,80,81,82,83,84,85,86,87,88,89,90,91,92,93,94,95,96,97,98,99,100,101,102,103,104,105,106,107,108]. As summarized in Table 1, these approaches have expanded beyond the conventional T-cell-based therapies to include alternative effector cells (e.g., NK cells, macrophages), as well as a broad range of engineering strategies, from ex vivo generation to in vivo gene delivery processes. In parallel, increasingly complex in vivo models have been employed to better recapitulate the tumor microenvironment and various combination strategies have been explored. Collectively, these studies provide critical insights into efficacy, safety and translational potential of FAP-targeted CAR therapies (Table 1).

Early investigations primarily relied on monolayer in vitro models as well as syngeneic and xenograft mouse models. More recent studies have increasingly incorporated advanced models, including 3D spheroid and organoid in vitro systems, as well as orthotopic or patient-derived xenograft (PDX) models, to better replicate the tumor microenvironment and stromal interactions, thereby potentially enhancing the predictive value for clinical translation (Table 1). So far, preclinical studies have predominantly employed second-generation CAR constructs incorporating either CD28 or 4-1BB costimulatory domains in combination with the CD3ζ signaling domain (Table 1). In recent years, however, more advanced designs have been increasingly explored, with several groups developing third-generation [87,97,101,108] and fourth-generation FAP CAR T-cells [81,90,96] to further enhance persistence, functionality, and therapeutic efficacy. Beyond signaling architecture, the preclinical studies in Table 1 also differ in their gene delivery strategy. Most constructs were introduced by retroviral or lentiviral vectors, which mediate stable integration and durable CAR expression. Lentiviral vectors, which transduce non-dividing cells, predominate among the more recent studies. In contrast to these stable expression approaches, non-integrating approaches have also emerged, notably CAR-encoding mRNA delivered by lipid nanoparticles [80,94,104] (Table 1). This confers transient expression that limits persistence and may thereby reduce side effects related to over-activation of CAR effector cells, although on-target/off-tumor toxicity arising from uptake by non-target cells remains a concern.

Across multiple studies, FAP CAR T-cells consistently reduced tumor growth by targeting CAFs, leading to remodeling of the tumor microenvironment and enhanced immune infiltration (Table 1). Schuberth and colleagues were among the first groups to demonstrate the therapeutic potential of targeting FAP-expressing cells. The authors employed second-generation anti-FAP-F19 CAR T-cells incorporating ΔCD28/CD3ζ domains, which effectively mediated in vitro killing of FAP-positive fibrosarcoma and mesothelioma cell lines. In intraperitoneal FAP-positive recombinant tumor xenograft models, FAP CAR T-cells significantly delayed tumor outgrowth and prolonged survival [100].

To further enhance therapeutic efficacy, several studies have explored CAR-based approaches that simultaneously target tumor cells and the tumor stroma (Table 1) [82,83,88,91,98,99,105,106,108]. Such strategies aim to overcome tumor heterogeneity and improve tumor eradication by targeting both malignant cells and their supportive microenvironment. Preclinical data indicate that these approaches can enhance antitumor activity and prolong survival compared with single-targeting strategies. On the one hand, simultaneous targeting can be achieved by combining FAP-targeted and tumor antigen-specific CAR T-cells, as demonstrated by Kakarla and colleagues in 2013 (Table 1). The authors successfully combined FAP- and EphA2-specific CAR T-cells in EphA2-positive A-549 xenograft mice, resulting in enhanced antitumor activity and prolonged survival compared with either monotherapy [88]. On the other hand, simultaneous targeting of both tumor and its stroma can be achieved by engineering bispecific CAR T-cells capable of recognizing two antigens with a single tandem receptor [98,108] or through distinct co-expressed receptors [83,99], or capable of secreting FAP-specific T-cell engagers [79,105] (Table 1).

In addition to CAR-based co-targeting approaches, several preclinical studies have investigated the combination of FAP-targeted CAR T-cells with other treatment modalities, including chemotherapy, cancer vaccines and immune checkpoint inhibitors (ICIs) [79,86,90,91,94,103,104,106]. These combinatorial strategies aim to further potentiate antitumor responses by modulating the tumor microenvironment, enhancing immune activation, or sensitizing target cells to CAR T-cell-mediated killing. Overall, such approaches have demonstrated improved therapeutic efficacy in preclinical models compared with monotherapies (Table 1).

In addition, several CAR T-cell strategies aiming to reduce immunogenicity and improving safety have been developed to enable allogeneic, off-the-shelf applications. Notably, Das et al. and Dharani et al. engineered non-alloreactive second-generation CAR T-cells using TALEN-mediated multiplex gene editing [82,83]. The resulting effector cells exhibited downregulation of surface TCRα/β and HLA-ABC. The combination of allogeneic FAP-targeted and mesothelin-specific CAR T-cells, together with PD-1 blockade, demonstrated significant antitumor activity and prolonged survival in an orthotopic xenograft mouse model of triple-negative breast cancer [82]. Furthermore, the use of NK cell and invariant NKT cells have also been explored as potential alternative cell types for off-the-shelf applications [81,84,97] (Table 1).

While most studies have employed ex vivo-engineered viral CAR T-cells, which demonstrate potent targeting of FAP-expressing cells, several groups have explored the in vivo generation of CAR effector cells [80,94,104] (Table 1). This approach offers potential advantages, including a faster and simplified manufacturing process, reduced costs, and the preservation of T-cell functionality compared with ex vivo manipulation. However, important challenges remain, particularly regarding safety (on-target/off-tumor effects), transfection efficiency, and (pre)clinical efficacy.

Comparing all the preclinical FAP-CAR studies available to date reveals that second-generation constructs bearing 4-1BB costimulatory domain predominate, reflecting a preference for slower effector response, greater persistence and lower exhaustion compared to CD28-bearing CAR T-cells [109]. Overall, most studies deliver effector cells by systemic intravenous adoptive transfer (Table 1), while the field has progressively diversified toward alternative effectors (macrophages, NK and iNKT cells) [81,84,97,104], in vivo mRNA-based CAR generation [80,94,104], and FAP/tumor-antigen co-targeting [82,83,88,91,98,99,105,106,108], which consistently improves single-targeting in the models tested.

#### 4.1.2. Safety Concerns

Despite promising efficacy in many preclinical studies, safety and toxicity remain central concerns in the development of FAP-directed CAR T-cell therapies. In addition to demonstrating limited to modest antitumor activity in vivo, Tran and colleagues reported that third-generation (CD28/4-1BB/CD3ζ) anti-murine FAP5-CAR T-cells induced substantial bone marrow toxicity and cachexia, which was attributed to the lysis of FAP-positive bone marrow stromal cells in mice (Table 1) [101]. Similarly, Wang et al. showed enhanced antitumor activity of KIR-based anti-murine FAP-73.3-CAR T-cells compared with second generation CD3ζ-based CAR T-cells in mesothelioma xenograft models. However, this potent effect was associated with substantial side effects such as anemia, hypocellularity of the bone marrow, and weight loss (Table 1) [102]. These observations underscore the importance of careful safety evaluation when using conventional FAP-targeted CAR T-cell strategies.

Importantly, the reasons underlying the heterogeneous toxicity profiles observed across the FAP CAR preclinical studies remain incompletely understood. Differences in CAR construct design (CAR generation, costimulatory domains selected), target affinity, and FAP specificity or cross-reactivity likely contribute to these differences. A major limitation may lie in the use of species-specific targeting antibodies: many early CAR constructs were directed against human FAP and evaluated in xenograft models (e.g., [82,100,108]), thereby preventing accurate assessment of on-target/off-tumor toxicity. Even though cross-reactive CARs (e.g., FAP-MO36, FAP-4G5) may partially address this limitation, intrinsic differences in FAP expression patterns and biology between mice and humans limit translational interpretation [24]. Furthermore, the diversity of experimental designs hinders direct comparability across studies and limits the identification of optimal therapeutic strategies. Overall, these challenges highlight the need for more physiologically relevant and standardized preclinical models to enable reliable prediction of safety profiles and to better inform clinical translation. Until such models exist, the heterogeneity of FAP antibody clones, tumor models, and toxicity readouts across studies only allows general design trends to be described, but prevents any reliable comparison of individual FAP-CAR strategies in terms of efficacy or safety.

Collectively, these preclinical studies evaluating FAP CAR T-cells highlight that therapeutic outcomes are not only driven by CAR design but also by effector cell type, engineering strategy, tumor model, and combinatorial approach. Insights from these investigations have guided the design of clinical trials and underscore the importance of carefully balancing efficacy and safety in the development of FAP-targeted CAR therapies.

### 4.2. Emerging Clinical Evidence

Several preclinical studies have enabled the clinical translation of FAP-targeted CAR T-cell therapy and the initiation of two phase I clinical trials as of 2026 (Table 2). Following the clinical protocol published by Petrausch and colleagues in 2012, as well as the preclinical studies conducted by Gulati et al. and Schuberth et al., the first clinical trial investigating the safety and persistence of second-generation F19 FAP CAR T-cells in patients with malignant pleural mesothelioma with pleural effusion was initiated in 2015 (NCT01722149, Table 2) [86,100,110]. Although no impact of the treatment on patient outcome could be evaluated due to the small number of patients, no CAR-related toxicities (e.g., CRS) were observed and CAR T-cell persistence was detected in peripheral blood [111,112,113].

In addition, based on the preclinical work performed by Li et al., a second clinical trial investigating the safety and tolerability of fourth-generation Nectin4/FAP CAR T-cells in patients with Nectin4-positive malignant tumors was initiated in 2019 (NCT03932565, Table 2) [90]. Although adverse events such as hemorrhagic rash and rash desquamation were reported (likely due to high Nectin4 expression in skin tissues), no severe CAR-related CRS or neurotoxicity was observed.

Overall, the preliminary results from these early-phase clinical studies are encouraging with respect to safety. These trials nevertheless remain small (four patients treated in NCT01722149 and an estimated enrolment of 30 in NCT03932565) and both employed loco-regional or intratumoral rather than systemic administration (as most preclinical studies did (Table 1)). The absence of severe toxicity in this setting therefore cannot be extrapolated to systemically administered FAP CAR T-cells, and the limitations discussed above (Section 4.1.2), including divergent constructs, antibody clones, and models, prevent direct comparison between these candidates and the broader preclinical literature. Additional clinical trials will be essential to validate the safety and evaluate the efficacy of CAR T-cell therapies in clinical applications.

## 5. Integration of FAP-Targeted Theranostics with Adapter CAR T-Cell Platforms

Over the past decades, multiple imaging approaches have been developed to improve tumor detection, disease staging and treatment monitoring. Among these, ^18^F-fluorodeoxyglucose (FDG) positron emission tomography (PET) has served as the main clinical standard. As FDG mirrors glucose metabolism and uses the enhanced glycolytic activity of tumor cells (Warburg effect), it provides robust tumor detection across various cancers. Despite clinical success, FDG faces limitations, including low metabolic uptake in some tumor entities, as well as uptake in normal, infectious and inflammatory tissues, which can lead to reduced specificity and tumor-to-background contrast [114,115]. These limitations have encouraged the implementation of complementary/alternative imaging strategies targeting specific antigen markers such as FAP.

In parallel, the concept of theranostic has emerged, integrating diagnostic imaging and targeted therapy within a single molecular approach. These strategies rely on the principle that the same molecular target can be used for both tumor imaging and therapeutic application, generally by using identical or (bio)chemically related tracers labeled with diagnostic or therapeutic radioisotopes [116,117]. This strategy enables patient selection, staging, treatment planning, therapeutic monitoring, personalized dose optimization based on target expression, as well as improved precision, as therapy is only administered to patients with tumors with optimal target expression [117]. The regulatory approval of ^177^Lu-DOTATATE (Lutathera) for the treatment of somatostatin receptor-positive neuroendocrine tumors and ^177^Lu-vipivotide tetraxetan (Pluvicto) for PSMA-positive metastatic prostate cancer in the last decade has stimulated the expansion of theranostic approaches [117].

Given its high expression in the tumor stroma of numerous solid tumors and its minimal expression in most normal adult tissues, FAP has emerged as a promising molecular target for theranostic applications [18,23]. Following the development of FAP-targeted theranostic tracers, adapter systems have increasingly incorporated these molecules for immunotheranostic purposes [25,26,27]. This review focuses on antibody- and small molecule-based approaches that were initially developed for theranostic targeting of FAP and have subsequently been integrated into adapter CAR T-cell systems.

### 5.1. FAPI-Based Imaging and Radioligand Therapy of the Tumor Microenvironment

#### 5.1.1. FAP-Directed Antibody Tracers

FAP-targeted radioligand delivery was first described in 1994, with the development of the FAP-specific murine monoclonal antibody (mAb) F19 [118]. Two phase-I dosimetry and distribution studies with iodine-131 (^131^I)-labeled mAb-F19 were performed in patients with hepatic metastases from colorectal carcinoma, or soft tissue sarcoma [118,119]. Both studies demonstrated proof of concept for the targeting of FAP-positive stromal cells, thereby encouraging further clinical investigation and development. Based on these results and aiming to reduce immunogenicity of the murine mAb-F19, Scott and colleagues developed the humanized mAb-F19, sibrotuzumab [120]. The authors evaluated safety, immunogenicity, pharmacokinetics, biodistribution, and tumor uptake of repeated injections of ^131^I-labeled sibrotuzumab in a phase I open-label dose escalation study including patients with colorectal carcinoma or non-small-cell lung cancer. Despite successful stromal targeting, treatment failed to provide objective tumor response and several patients were removed from the study after developing human anti-human antibodies [120]. Further clinical development of sibrotuzumab was ultimately discontinued.

In an effort to address the limitations observed with the F19 mAb, Fischer and colleagues developed two novel human/murine cross-reactive anti-FAP human mAbs, ESC11 and ESC14, via phage display. Notably, the authors demonstrated that the ^177^Lu-labeled antibodies selectively accumulated in FAP-positive melanoma xenograft models, thereby delaying tumor outgrowth and extending mouse survival [121]. Since then, several research groups have developed and evaluated new FAP-directed antibody-based radiotracers for theranostic purposes in preclinical models of glioblastoma [122], prostate cancer [123], fibrosarcoma [124] and breast cancer [125]. Overall, these studies have demonstrated encouraging results; however, further evaluation of pharmacokinetics, immunogenicity, toxicity, and efficacy remains necessary to enable clinical translation.

#### 5.1.2. FAP-Directed Small Molecule-Based Tracers

The design of small-molecule FAP inhibitors has dramatically facilitated the development of FAP-targeted theranostic approaches [23,24]. To date, small-molecule FAPI tracers have gained increasing clinical interest due to their rapid imaging capabilities, relatively fast in vivo clearance, and generally high tumor-to-background contrast compared with antibodies [21,22]. The first generation of FAP inhibitors, designed using dipeptide boronic acid scaffolds, had poor selectivity towards FAP over DPPs and prolyl oligopeptidase (PREP) [23,24]. In 2013, several highly potent and selective FAPI compounds based on quinolines, such as UAMC-1110, were introduced by Jansen and colleagues [126,127].

To enable radioligand delivery applications, monomeric quinoline-based FAPIs were subsequently modified to incorporate radiohalogens (e.g., iodine-125) or chelators (e.g., DOTA) for labeling with radioisotopes such as gallium-68 (^68^Ga) [128]. For the first time, the authors demonstrated robust tumor accumulation of the ^68^Ga-labeled tracer FAPI-02 in patients with metastasized breast cancer, lung cancer or pancreatic cancer, while showing low tracer uptake in healthy tissues [128]. However, FAPI-02 exhibited fast clearance, with tumor uptake dramatically decreasing between 1 and 3 h post-injection, thereby limiting its use for therapeutic applications. Aiming to improve tumor retention time, Lindner and colleagues developed variants of FAPI-02, leading to the generation of several novel tracers [129]. Among these, FAPI-04 showed promises both in vitro and in vivo: PET imaging of patients with metastasized breast cancer injected with ^68^Ga-FAPI-04 demonstrated rapid and high tracer uptake in metastases. In addition, treatment with FAPI-04 labeled with the therapeutic nuclide yttrium-90 (^90^Y) led to a reduction in pain, encouraging the further development of such FAPI radiotracers for both nuclear imaging and therapy [129]. The same group further designed FAPI radiotracers to increase tumor uptake and retention, leading to the generation of FAPI-46 [130]. Compared to ^68^Ga-FAPI-04, ^68^Ga-FAPI-46 displayed improved tumor retention and tumor-to-background ratio [130]. To date, ^68^Ga-FAPI-04 and ^68^Ga-FAPI-46 are broadly used for PET imaging of various solid tumors and being investigated in numerous phase I and II clinical trials (e.g., NCT05160051, NCT05898854, NCT05003427, NCT07132645, NCT06790082).

Although monomeric FAP inhibitors demonstrated success for imaging of numerous human tumor entities [131,132], retention time of such monomeric tracers remains limited (a few hours), which may limit their use as FAP-specific radiotherapeutic agents [23,24,132,133]. In this context, the tumor retention half-life of a radiotracer should ideally match the physical half-life of the selected radionuclide to maximize radiation dose at the tumor site while minimizing potential side effects associated with radiotracer clearance [132]. To enhance tumor uptake and prolong retention time, several strategies have been developed [24], including the use of multimeric FAPI moieties within a single radiotracer (e.g., [134,135,136,137]), the incorporation of albumin-binding motifs (e.g., [138,139,140,141]), warhead optimization of UAMC-1110 [142], linker modifications (e.g., [143,144]) and the development of covalent-targeted radioligands [145].

Overall, FAP-based imaging has demonstrated pan-tumor utility, high tumor-to-background contrast and sensitivity, rapid tumor uptake, as well as accurate disease staging in several studies [62]. Despite these encouraging results, several challenges remain, including the optimization of tracer pharmacokinetics, minimization of uptake in healthy tissues, and long-term evaluation of therapeutic efficacy. Complementary approaches, such as immunotherapeutic strategies, may offer the potential to actively reshape the tumor microenvironment and improve clinical outcomes. In this context, adapter CAR systems represent a particularly suitable platform, as they enable the integration of radioligand delivery with CAR T-cell immunotherapy while providing enhanced safety through controlled and modular targeting.

### 5.2. FAP-Specific Molecules for Adapter CAR T-Cell Therapy and Radioligand Delivery Applications

Beyond CAR T-cell redirection, the concept of immunotheranostics has emerged, extending the traditional theranostic concept toward the integration of radioligand delivery and immune-based therapies [41]. In this context, nuclear imaging enables the assessment of target expression and accessibility, as well as monitoring of treatment response. Modular adapter CAR systems represent a highly valuable platform for such applications as they decouple antigen recognition from CAR T-cell activation, allowing identical or chemically related adapter molecules to be used for both imaging and therapy.

Based on the potential of FAP-targeted CAR T-cell therapies and radioligand delivery applications, several groups have developed and characterized FAP-specific adapter molecules for adapter CAR T-cell immunotherapy and/or theranostic approaches [25,26,27,146] (Table 3). Huang and colleagues designed a FAPI-based adapter to redirect FITC-directed modular CAR T-cells [146]. This construct comprises the FAP-specific small-molecule ligand FAP8 connected to fluorescein via a 18-unit polyethylene glycol (PEG_18_) spacer. By combining folate- and FAP-specific adapters in tumor xenograft models, the authors demonstrated enhanced tumor growth inhibition when modular CAR T-cells simultaneously targeted folate-positive tumor cells and FAP-positive fibroblasts. This dual-targeting approach improved CAR T-cell proliferation, activation, tumor infiltration, and intratumoral distribution without inducing significant toxicity [146] (Table 3).

Millul and colleagues developed an adapter molecule based on the UAMC-1110 inhibitor, capable of redirecting adapter CAR T-cells to FAP-expressing cells (Table 3) [27]. This adapter consists of the OncoFAP moiety, which specifically binds to FAP, and a fluorescein dye recognized by fluorescein-directed adapter CAR T-cells. They demonstrated efficient in vitro elimination of the recombinant cell line SK-RC-52.hFAP by anti-FITC CAR T-cells, which is strictly dependent on the presence of the FAP-specific adapter (Table 3). In parallel, Millul et al. developed another OncoFAP derivative which, when radiolabeled with lutetium-177, selectively accumulated in recombinant tumor-bearing mice and exhibited a favorable biodistribution profile.

Our group recently developed antibody-based adapter molecules for the UniCAR system derived from the humanized anti-FAP F19 monoclonal antibody and incorporating the E5B9 UniCAR epitope (Table 3) [26,148]. These target modules mediated significant tumor growth inhibition in vivo and, upon conjugation to NODA-GA and radiolabeling with copper-64, enabled selective accumulation at FAP-positive tumor sites for non-invasive imaging [26]. Both the approaches established by Millul et al. and Loureiro et al. enabled adapter CAR T-cell therapy and delivery of radioligand while respectively relying on chemically non-identical but related adapter molecules for each application. Notably, the use of distinct molecules for CAR T-cell redirection and radioligand delivery, including chelator-modified variants, may result in distinct pharmacokinetic profiles.

In a recent study, our group converted a small molecule-based PET tracer directed to the prostate membrane antigen (PSMA) into an immunotheranostic TM for the UniCAR system [41]. After extensive preclinical evaluation, it was even translated into a clinical phase 1 trial (NCT04633148). The tracer-based TM was well tolerated but the clinical efficacy was low, which was most likely caused by a very rapid elimination of the theranostic TM. Building on this experience, we aimed to develop a single molecule for both radioligand delivery applications and adapter CAR T-cell therapy directed against FAP. The FAPI-based UniCAR TMs consist of a monomeric FAPI structure based on the UAMC-1110, which was chemically fused to the E5B9 UniCAR epitope, a PEG spacer between the inhibitor and the epitope, and the chelator NODA-GA, enabling radiolabeling with diagnostic and therapeutic radionuclides (Table 3) [25]. So far, we demonstrated that spacer optimization (PEG_12_/PEG_24_) enables potent redirection of UniCAR T-cells to FAP-positive recombinant cells in vivo. In addition, copper-64-labeled FAPI TMs selectively accumulated in FAP-positive tumors in vivo, highlighting the potential of the UniCAR system for both non-invasive diagnostic imaging and adapter CAR T-cell immunotherapy through the use of a single adapter molecule. Aiming to optimize the pharmacokinetic properties of the monomeric FAPI UniCAR TMs, particularly for radiotherapeutic applications, we further developed a FAPI homodimer consisting of two UAMC-1110 moieties fused to the E5B9 peptide by a PEG spacer, and a functional group that allows for future TM functionalization for radioligand delivery applications [148]. These homodimeric FAPI TMs promoted potent UniCAR-mediated lysis of FAP-positive cells both in vitro and in an immunodeficient mouse model [148]. In addition, combining homodimeric FAPI and anti-PSCA TMs enhanced UniCAR-mediated cytotoxicity against heterotypic spheroids composed of FAP-positive fibroblasts and PSCA-positive tumor cells.

Comparing the FAP-directed adapters reported to date (Table 3) reveals that they differ in affinity, size, and clearance, with direct consequences for their diagnostic and therapeutic use. On the one hand, while antibody-based adapters offer high affinity and long circulation [26,148], their slow clearance may be poorly matched to short-lived radionuclides, and the FAP-specific F19 lineage raised immunogenicity concerns in patients [110]. On the other hand, monomeric FAPI-based adapters derived from UAMC-1110 clear rapidly and deliver high tumor-to-background contrast within hours, which suits imaging but limits the absorbed dose in the case of radioligand therapeutic applications [25,27]. Matching tumor residence time of the adapter and the physical half-life of the diagnostic and therapeutic radionuclide is therefore a central parameter [24]. Strategies that extend residence time, such as multimerization [134,135,136,137], introduction of albumin binding [138,139,140,141], linker and warhead optimization [142,143,144], and covalent targeting [145], may address this mismatch. However, the resulting longer circulation carries its own cost, raising background signal and, for adapter CAR systems in particular, extending the interval during which CAR activity cannot be switched off. This parameter distinguishes FAP-targeted immunotheranostic adapters from conventional radiotracers, since the pharmacokinetics of the adapter controls not only the radiation dose delivered at the tumor site but also the duration of adapter CAR T-cell activation. One way to escape this dual constraint is a pre-targeting approach, in which the adapter is allowed to accumulate at the tumor site and clear from the circulation before a separately administered radionuclide is delivered [149]. Decoupling the two steps would allow the adapter to be optimized for CAR redirection and radioligand delivery independently. Adapter molecules designed to support both CAR T-cell redirection and pre-targeted radioligand delivery may therefore represent a particularly attractive route toward integrated immunotheranostic platforms in the future.

Beyond the design of individual adapters, the broader relationship between FAP-directed radioligand delivery and FAP-targeted adapter CAR T-cell therapy is worth considering, as the two modalities may interact in both beneficial and counterproductive ways. On the one hand, FAP-directed PET imaging enables patient selection and response monitoring, and has already been used to track anti-FAP CAR T-cell activity in mouse models [89]. Radioligand-mediated depletion of FAP-positive stromal and tumor tissues may, in principle, reshape the TME by softening the extracellular matrix, reducing immunosuppression and thereby improving the infiltration of subsequently infused CAR T-cells, an effect demonstrated for CAR-mediated stromal depletion [106]. However, this remains a conceptual approach that has not been experimentally tested. On the other hand, the two modalities may interfere. Because both act on the same FAP-positive compartment, radioligand-mediated CAF depletion may reduce the antigen density available for subsequent adapter-mediated redirection, while radiation-induced lymphopenia may compromise CAR T-cell engraftment and persistence. The sequencing, timing, and dose fractionation of the two approaches therefore represent an essential, and currently unresolved, question for the rational design of FAP-directed immunotheranostic strategies.

Overall, the dual functionality of adapter CAR systems expands their translational potential and highlights new opportunities to integrate diagnostic and therapeutic strategies within a single immunotheranostic platform, although all evidence for FAP-directed immunotheranostic adapters remains preclinical. Further preclinical studies and clinical trials will be required to (1) assess the safety and efficacy of FAP-targeted adapter CAR T-cell therapies in solid tumors, including comprehensive evaluation of pharmacokinetics, immunogenicity, and optimal dosing regimens; (2) determine the feasibility and clinical benefit of combination strategies designed to simultaneously target tumor cells and the TME with different adapters; (3) assess the potential of adapter molecules to enable theranostic strategies, including diagnostic or radiotherapeutic applications and real-time monitoring of therapeutic response during treatment; and (4) define the optimal sequencing and dosing of radioligand therapy and adapter CAR T-cell therapy when the two are combined.

## 6. Conclusions and Future Perspectives

Due to its overexpression in numerous solid tumor types and its key role in tumor progression, FAP has emerged as an attractive target for both the diagnosis and treatment of solid malignancies. As summarized in this review, the last decade has witnessed a rapid expansion of preclinical studies showing that FAP-directed CAR T-cells can selectively and effectively kill target cells in vitro and in vivo, leading to the clinical translation of two candidates. In parallel, FAP-targeted radiotracers, particularly those based on FAP inhibitors, have gained increasing interest and shown promise in the theranostic management of solid tumors. To our knowledge, this is the first review to consider FAP-directed CAR T-cell therapy and FAP-directed theranostics together rather than as separate fields. By covering conventional and adapter-based FAP-CAR strategies alongside FAP-targeted radioligand delivery, it provides an updated picture of the field and shows how modular adapter platforms bring immunotherapy and nuclear medicine into a single, unified immunotheranostic framework.

Despite this progress, safety remains the most important consideration for its translation. Several preclinical studies have reported significant on-target/off-tumor effects, such as bone marrow toxicity and cachexia, whereas many others, using distinct scFv clones, costimulatory domains and tumor models, observed potent antitumor activity without obvious toxicity. The origin of these distinct safety profiles remains unclear as most preclinical studies differ in CAR design, FAP-directed antibody clone, tumor model, and toxicity readout, which prevents direct comparison. Moreover, several studies reporting no toxicity lacked models able to fully evaluate it. These challenges highlight the need for more physiologically relevant and standardized preclinical models to predict safety more reliably, identify optimal therapeutic strategies, and guide clinical translation. Another concern is the soluble form of FAP present in the plasma of healthy individuals and patients, which raises the theoretical risk of off-tumor effects, complicating the prediction of the accumulation and excretion/elimination of FAP-directed agents [59,60]. Accounting for soluble FAP is therefore an important consideration in the design of radiotracer- and immunotherapy-based FAP-directed clinical strategies. Reassuringly, FAP-directed radioligand applications have demonstrated relatively high tumor-to-background ratios [21,62] and the FAP-targeted CAR T-cell therapies evaluated clinically to date have shown favorable safety profiles, with both phase I trials reporting no severe treatment-related toxicity [90,111,112,113]. The effect of soluble FAP has nevertheless not been formally quantified and clinical experience remains limited: larger controlled trials are needed to confirm both efficacy and safety.

Given that FAP targeting alone is unlikely to achieve complete tumor eradication in solid malignancies, therapeutic success will most likely rely on combinatorial approaches that address both tumor cells and the tumor microenvironment, integrating CAR T-cell therapy, targeted radioligand therapy, and other clinically relevant modalities. In this context, the convergence of advances in FAP-directed CAR T-cell therapy and FAP-targeted theranostic agents has given rise to modular approaches based on adapter CAR systems, which offer improved specificity, target flexibility, and controllability, while enabling the integration of FAP-specific CAR T-cell immunotherapy and radioligand delivery within a unified framework. Their switchable and dose-dependent control of CAR activity may mitigate the toxicities associated with conventional CAR constructs. So far, they have yielded encouraging preclinical results for immunotheranostic targeting of FAP in solid tumors [25,26,27,146].

Further progress in the field will depend on several factors. First, more standardized and physiologically relevant safety models, such as humanized or syngeneic mouse models with a particular focus on bone marrow toxicity, should be adopted so that on-target/off-tumor risk can be compared meaningfully across CAR systems. Accounting for intrinsic differences in FAP expression patterns and biology between preclinical models and humans will also be critical to the clinical translation of FAP-targeted therapies. Second, systematic characterization of FAP expression across CAF subsets and tumor entities will be needed to identify the tumor types in which FAP-positive CAFs are predominantly tumor-promoting and therefore suitable for depletion. Third, the pharmacokinetics of FAP-directed immunotheranostic adapters must be optimized to satisfy two requirements: the residence time needed for diagnostic sensitivity and/or radiotherapeutic efficacy, and the clearance needed for switchable control of adapter CAR activity [24]. Fourth, FAPI-based tracers will likely play a key role in guiding FAP-directed CAR T-cell therapy, enabling patient selection, dose determination, and monitoring of therapeutic response in addition to tumor imaging, an advantage that distinguishes FAP from most other CAR targets in solid tumors. Fifth, therapeutic success will most probably rely on the rational combination of FAP-targeted agents with TAA-directed CARs and other modalities, given that FAP targeting alone is unlikely to eradicate solid tumors. Finally, although FAP-targeting immunotheranostic adapter systems have shown encouraging results in preclinical models, further preclinical validation and early clinical studies are required to demonstrate their safety and efficacy, and a conceptual framework is needed to define how these strategies can be optimally implemented in cancer patients. Their clinical translation will specifically require optimal trial design, with particular attention to adapter dosing and scheduling, FAP-directed imaging endpoints, and the still-unaddressed regulatory question of whether a single CAR T-cell product can be approved for use with multiple adapters. Collectively, while these approaches remain at an early stage and must still address important limitations, they highlight the potential of FAP-directed immunotheranostic strategies as a promising approach for next-generation solid tumor diagnosis and treatment.

## Figures and Tables

**Table 1:** Preclinical landscape of FAP-targeted CAR T-cell therapies in cancer.

Type of Cancer	CAR Construct (ICDs), Reactivity, Route of In Vivo Administration	Model(s)	Outcome	Ref.
Melanoma, colon, breast, fibrosarcoma, kidney, PDAC	3rd gen retroviral FAP5-CAR (mCD28/m4-1BB/mCD3ζ), hu./mu, i.v.	In vitro + syngeneic (target stromal FAP) and xenograft mice	Limited to modest efficacy; severe BM toxicity /cachexia	Tran et al., 2013 [101]
Lung, prostate, melanoma, glioblastoma	2nd gen retroviral FAP-MO36 CAR (CD28/CD3ζ), hu./mu., i.v.	In vitro + xenograft mice (target stromal FAP)	Combination of FAP CAR + EphA2 CAR T-cells improved antitumor efficacy and survival; no toxicity	Kakarla et al., 2013 [88]
Malignant pleural mesothelioma	2nd gen retroviral FAP-F19 CAR (ΔCD28/CD3ζ), hu., i.p.	In vitro + xenograft mice (target tumor FAP)	Significant antitumor activity & improved survival; led to Phase I trial NCT01722149	Schuberth et al., 2013 [100]
Mesothelioma, lung, colon, breast	2nd gen retroviral FAP-73.3 CAR (4-1BB/CD3ζ), mu., i.v.	In vitro + syngeneic & immunodeficient mice (target stromal FAP)	Tumor reduction via FAP^+^ cell depletion; enhanced by 2nd dose or combination w. cancer vaccine; min. toxicity	Wang et al., 2014 [103]
Mesothelioma, lung, PDAC	2nd gen retroviral FAP-73.3 CAR (4-1BB/CD3ζ), mu., i.v.	In vitro + syngeneic and xenograft mice (target stromal FAP)	Stromal FAP^+^ depletion reduced ECM/vasculature density and inhibited the growth of desmoplastic tumors	Lo et al., 2015 [92]
Malignant mesothelioma	FAP-73.3-KIRS2 lentiviral CAR (KIR2DS2 TM and cytoplamic domain) in T-cells expressing DAP12, mu., i.v.	Xenograft mice (target stromal FAP)	Enhanced antitumor activity and FAP^+^ cell depletion vs. conventional CAR; toxicity observed (e.g., anemia, weight loss)	E. Wang et al., 2015 [102]
Malignant pleural mesothelioma	2nd gen retroviral FAP-F19 CARs (CD28/CD3ζ, ΔCD28/CD3ζ or 4-1BB/CD3ζ), hu., i.p.	In vitro + xenograft (humanized mice, target tumor FAP) + First-in-man (NCT01722149)	ΔCD28 CAR showed improved tumor control with PD-1 blockade in mice; persistence up to 21 days in MPM patient	Gulati et al., 2018 [86]
Lung cancer	4th gen lentiviral FAP-12 CAR (4-1BB/CD3ζ) with constitutive expression of IL-12, hu./mu., i.v.	In vitro + xenograft mice (target stromal FAP)	Combi. of Nectin4-7.19 and FAP-12 CAR T-cells prolonged survival vs. controls; no toxicity; led to phase I trial (NCT03932565)	Li et al., 2022 [90]
Lung cancer	2nd gen retroviral FAP-4G5 CAR (4–1BB/CD3ζ), hu./mu., i.v.	In vitro + xenograft mice (target stromal FAP)	Significant reduction in tumor volume, FAP^+^ cells deletion monitored by [^18^F]-AlF-FAPI-74 non-invasive PET imaging	Lee et al., 2022 [89]
Hepatocellular carcinoma	3rd gen lentiviral FAP CAR (CD28/TLR2/CD3ζ), i.v.	In vitro + xenograft mice	FAP CAR T-cells promote significant tumor suppression in induced HCC mice	Jiang et al., 2022 [87]
Multiple myeloma	2nd gen BCMA-C11D3 (4-1BB CD3ζ, hu.) and FAP-05 (4-1BB/CD3ζ, hu./mu.) lentiviral dual CAR T-cells, n.m.	In vitro + xenograft mice (target stromal FAP)	Dual CAR outperformed BCMA CAR with prolonged survival in MM xenografts with BM-CAFs	Sakemura et al., 2022 [99]
PDAC	2nd gen retroviral FAP-8E3 CAR (4-1BB/CD3ζ), hu./mu., i.v.	In vitro + allograft mice (target stromal FAP)	FAP + Claudin 18.2 CAR T-cell combination enhanced activity (CAF depletion and reduced MDSC recruitment); no toxicity	Liu et al., 2023 [91]
TNBC	2nd gen lentiviral FAP-hF1 UCAR (hu.) and FAP-mF3 UCAR (hu./mu) (4-1BB/CD3ζ) in TALEN-edited non-alloreactive T-cells, i.v.	In vitro + orthotopic xenograft mice (target stromal FAP)	FAP-hF1 UCAR depleted CAFs, restoring sensitivity to Meso UCAR T-cells; triple therapy w. anti-PD-1 reduced tumor burden and prolonged survival	Das et al., 2023 [82]
Non-small-cell lung cancer	2nd gen lentiviral hFAP CAR (4-1BB/CD3ζ) in NK-92 cells, hu., i.v.	In vitro + xenograft mice (target stromal FAP)	hFAP-CAR-NK-92 significantly inhibited tumor progression vs. NK-92 cells	Fang et al., 2023 [84]
PDAC with desmoplastic stroma	2nd gen retroviral FAP-4G5 CAR (4-1BB/CD3ζ), hu./mu./ca., i.v.	In vitro + syngeneic, autochthonous and xenograft mice (target stromal FAP)	FAP-CAR mediated CAF depletion and stromal remodeling; enhanced synergy w. mesothelin-CAR or PD-1 block.; min. toxicity	Xiao et al., 2023 [106]
PDAC	2nd gen lentiviral anti-mesothelin meso^FAP^ CAR-TEAM cells secreting FAP-specific TEAM (sibrotuzumab), hu., i.v.	In vitro (patient-derived spheroids and organoids) + xenograft mice (target tumor and stromal FAP)	Meso^FAP^ CAR-TEAM cells showed superior PDAC/CAF elimination vs. single targeting in 3D in vitro and mouse models; stromal remodeling improved tumor clearance	Wehrli et al., 2024 [105]
Hepatocellular cancer	3rd gen lentiviral tandem bispecific FAP-GPC3 CAR (CD28/TLR2/CD3ζ), hu., p.t.	In vitro + xenograft mice (including PDX) (target tumor FAP)	Bispecific CAR T-cells showed superior efficacy and survival vs. monospecific CAR; GVHD in PDX model	Zhou et al., 2024 [108]
Glioblastoma	2nd gen lentiviral FAP-MO36 CAR (CD28/CD3ζ), mu./hu., i.v.	In vitro + xenograft mice (target tumor FAP)	Tumor control via Ag-dependent killing of FAP^+^ and FAP^−^ (bystander effect) cells; no significant toxicity	Yu et al., 2024 [107]
Fibrosarcoma, glioblastoma, hepatic, pancreatic, breast cancers	2nd gen lentiviral FAP CAR (4-1BB/CD3ζ), mu./hu., i.v.	In vitro + allograft and xenograft (including PDX) (target tumor and stromal FAP)	FAP CAR T-cells showed potent in vivo killing of FAP^+^ cells/CAFs; no significant toxicity	Niu et al., 2024 [95]
PDAC	2nd gen retroviral FAP-h8E3 and claudin18.2-h8E5-2I-specific bispecific CARs (4-1BB/CD3ζ), mu./hu., n.m.	In vitro + allograft mice (target stromal FAP)	Bispecific CARs improved antitumor efficacy vs. conventional CARs (decreased MDSC recruitment and T-cell exhaustion); no significant toxicity	Sun et al., 2024 [98]
Breast, lung	TALEN-edited allogenic PD-1- inactivated T-cells expressing lentiviral 2nd gen FAP-hF1 CAR (4-1BB/CD3ζ), hu.; activation induces mesothelin CAR expression, i.v.	In vitro (including mono and hetero-spheroids) + xenograft mice (target tumor and stromal FAP)	Dual inducible CAR T-cells enhanced antitumor activity and survival vs. controls; no significant OTOT toxicity	Dharani et al., 2024 [83]
Colorectal	Adeno-associated virus 1st gen FAP-MO36 CARs (CD3ζ, ΔCD3ζ or FcRγ) in BMDMs, hu./mu., i.v.	In vitro *+* syngeneic mice (target stromal FAP)	Only FAP CAR-ΔCD3ζ BMDMs inhibited MC38 tumors via FAP^+^ CAF targeting and TAM reprogramming	Mao et al., 2024 [93]
Colorectal cancer	Lentiviral FAP CAR T-cells secreting PD-1 nanobody (FAP-PD-1/Nb CAR T)	In vitro	FAP–PD-1/Nb CAR T-cells showed superior immunity and infiltration vs. single-target CARs	Gao et al., 2025 [85]
PDAC	Mannose-modified LNPs loaded w. 1st gen FAP-73.3 CAR mRNA to reprogram M2 macrophages in vivo, mu., i.v.	In vitro + xenograft mice (target stromal FAP)	FAP-CAR macrophages inhibited CAFs, improved chemo./PD-1 efficacy and prolonged survival; no significant toxicity	Wang et al., 2025 [104]
Cervical cancer	3rd gen alpharetroviral FAP-ESC11 CAR (CD28/4-1BB/CD3ζ) in NK-92 cells and primary cord blood-derived NK cells	In vitro (including mono- & heterospheroids)	Significant cytotoxicity against FAP^+^ cervical cancer cells and primary CAFs	Polten et al., 2025 [97]
Colorectal, breast & renal cancers	LNPs loaded w. 2nd gen FAP CAR (4-1BB/CD3ζ) mRNA to reprogram immune cells in vivo (non-targeted), hu. or mu., i.v.	In vitro + xenograft mice (including PDX) (target stromal FAP)	Significant and durable antitumor activity in combination with other treatments (e.g., ICIs, chemotherapy)	Meng et al., 2025 [94]
Glioblastoma & liver cancer	4th gen lentiviral FAP CAR (4-1BB/CD3ζ) with constitutive expression of IL-15, hu., i.v.	In vitro + xenograft mice (including PDX) (target tumor and stromal FAP)	4th gen CAR enhanced efficacy, proliferation, memory (T_SCM_-like phenotype) vs. 2nd gen; min. toxicity	Pang et al., 2025 [96]
PDAC	2nd gen lentiviral anti-mesothelin meso^FAP^ CAR-TEAM (4-1BB/CD3ζ) T-cells secreting FAP-specific TEAM, hu., i.v. and i.p.	In vitro + xenograft mice (including PDX) (target stromal FAP)	Ibrutinib enhanced CAR T expansion & antitumor activity; PD-1 blockade improves CAR T-cell potency in PDX models	Armstrong et al., 2026 [79]
Melanoma, lung & colorectal cancers	4th gen lentiviral allogeneic MiNK-215 FAP CAR (4-1BB/CD3ζ) in iNKT-cells secreting IL-15, hu./mu., i.v.	In vitro (including spheroids and organoids) + xenograft mice (target stromal FAP)	Controlled tumors via TME remodeling and immune activation; minimal bone marrow toxicity	Chantzoura et al., 2026 [81]
PDAC	Anti-CD5 LNPs loaded w. 2nd gen FAP-73.3 CAR (CD28/ CD3ζ) mRNA to reprogram CD5^+^ immune cells in vivo, mu., i.v.	In vitro + xenograft mice (target stromal FAP)	In vivo CAR T-cells ≥ ex vivo retroviral CAR T-cells in tumor inhibition; no systemic toxicity	Bajbouj et al., 2026 [80]

CAR constructs are described by generation (gen) as defined in Section 2.1.1, along with intracellular signaling domains (ICDs, listed in parentheses), the FAP-directed binding clone (when reported), the vector system, species reactivity (hu., human; mu., murine; ca., canine), and route of administration of the CAR or mRNA/LNP product (i.p.: intraperitoneal injection; i.v.: intravenous injection; p.t.: peritumoral injection; n.m.: not mentioned). “Tandem” refers to two binding domains within one receptor; “dual” indicates two co-expressed receptors; “inducible” refers to activation-dependent expression of a second receptor. Abbreviations: Ag: antigen; BMDM: bone marrow-derived macrophage; ECM: extracellular matrix; HCC: hepatocellular carcinoma; ICI: immune checkpoint inhibitor; iNKT: invariant natural killer T; LNP: lipid-based nanoparticle; MDSC: myeloid-derived suppressor cell; min.: minimal; Nb: nanobody; OTOT: on-target/off-tumor; PDAC: pancreatic ductal adenocarcinoma; PDX: patient-derived xenograft; TALEN: transcription activator-like effector nuclease; TAM: tumor-associated macrophage; TM: transmembrane; TNBC: triple-negative breast cancer; T_SCM_: T memory stem cell.

**Table 2:** Clinical landscape of FAP-targeted CAR T-cell therapies in cancer.

Trial Design	Disease and Study Aim	Construct and Treatment Regimen	Main Outcomes	Ref.
NCT01722149, early phase 1, initiated in 2015, 4 participants	Malignant pleural mesothelioma with pleural effusion; safety and persistence of intra-pleural administered anti-FAP CAR T-cells in the periphery	2nd gen FAP-F19 CD3ζ/ CD28 CAR CD8^+^ T-cells; loco-regional administration of a single dose of 1 × 10^6^ CAR T-cells through a pleural catheter	No CAR T-cell related toxicities, persistence detected in peripheral blood	Curioni et al., 2019; Gulati et al., 2018; Hiltbrunner et al., 2021; Petrausch et al., 2012; Pircher et al., 2015; Schuberth et al., 2013 [86,100,110,111,112,113]
NCT03932565, Phase 1, initiated in 2019, 30 participants (estimated enrolment)	Advanced Nectin4+ malignant solid tumors; safety and tolerability of Nectin4/FAP CAR T cells in Nectin4^+^ tumors	4th gen Nectin4/FAP CAR T-cells expressing IL-7 and CCL19 or IL-12; intratumoral injection of Nectin4/FAP CAR T-cells	Hemorrhagic rash and rash desquamation due to Nectin4 expression in skin tissues, no CAR-related severe CRS or neurotoxicity	Li et al., 2022 [90]

**Table 3:** Design of FAP-specific adapters for modular CAR T-cell therapy or/and radioligand delivery in preclinical models.

Adapter CAR System	Features of FAP-Targeted Adapters	Proof of Concept	Ref.
Anti-fluorescein CAR system	**FAP8-fluorescein** small-molecule FAP8 coupled to fluorescein	In vivo redirection of adapter CAR T-cells by both folate- and FAP-specific adapters promotes significant tumor growth inhibition compared to single adapter administration (xenograft)	Huang et al., 2025 [146]
Anti-fluorescein CAR system	**OncoFAP-fluorescein** small-molecule OncoFAP (derived from UAMC-1110) coupled to fluorescein	In vitro CAR-mediated killing of SK-RC-52.hFAP cells in presence of OncoFAP-fluorescein	Millul et al., 2021 [27]
	**OncoFAP-DOTAGA** OncoFAP coupled to DOTAGA for ^177^Lu labeling	In vivo quantitative biodistribution: high, selective accumulation in FAP^+^ xenograft tumors	
UniCAR system	**⍺FAP-scFv-E5B9** and **⍺FAP-IgG4-E5B9 TMs** antibody derivatives fused to E5B9 peptide, require further chemical modification for radioligand delivery applications	In vivo immunotheranostic targeting: UniCAR + ⍺FAP TMs promote significant tumor inhibition (co-injection); ^64^Cu-NODA-GA-labeled TMs enable non-invasive diagnostic imaging of FAP^+^ xenograft tumors	Loureiro et al., 2023 [26]
	**NODA-GA-FAPI-PEG_12/24_-E5B9 TMs** UAMC-1110 coupled to E5B9 peptide and NODA-GA chelator, ready for immunotheranostic applications	In vivo immunotheranostic targeting: UniCAR + FAPI TMs promote significant tumor inhibition (co-injection); ^64^Cu-labeled TMs enable non-invasive diagnostic imaging of FAP^+^ xenograft tumors	Boutier et al., 2025 [25]
	**(FAPI)_2_-PEG_16/24_-E5B9** UAMC-1110 homodimer coupled to E5B9 peptide, require further chemical modification for radio- ligand delivery applications	In vivo redirection of UniCAR T-cells by homodimeric FAPI TMs promotes significant tumor growth inhibition (co-injection); homodimeric FAPI + anti-PSCA TMs promote synergistic killing of heterospheroids in vitro	Boutier et al., 2026 [147]

## Data Availability

No new data were created or analyzed in this study. Data sharing is not applicable to this article.

## Abbreviations

The following abbreviations are used in this manuscript:

FAP	Fibroblast activation protein alpha
CAR	Chimeric antigen receptor
Ag	Antigen
BMDM	Bone marrow-derived macrophage
Ca.	Canine
ECM	Extracellular matrix
HCC	Hepatocellular carcinoma
Hu.	Human
ICD	Intracellular domain
ICI	Immune checkpoint inhibitor
iNKT	Invariant natural killer T
LNP	Lipid-based nanoparticle
MDSC	Myeloid-derived suppressor cell
Mu.	Murine
PDAC	Pancreatic ductal adenocarcinoma
PDX	Patient-derived xenograft
PEG	Polyethylene glycol
TALEN	Transcription activator-like effector nuclease
TAM	Tumor-associated macrophage
TSCM	T memory stem cell
